# Data-centric strategies enable cross-center generalization of convolutional neural networks for CT brain extraction

**DOI:** 10.3389/fneur.2026.1895626

**Published:** 2026-08-03

**Authors:** Zheng Sun, Lifang Ye, Yao Xu, Yuehua Li

**Affiliations:** 1 School of Health Science and Engineering, University of Shanghai for Science and Technology, Shanghai, China; 2 Institute of Diagnostic and Interventional Radiology, Shanghai Sixth People’s Hospital Affiliated to Shanghai Jiao Tong University School of Medicine, Shanghai, China; 3 Department of Radiology, Liyang Hospital of Chinese Medicine, Changzhou, Jiangsu, China

**Keywords:** cross-center generalization, CT brain tissue extraction, deep learning, incremental fine-tuning, U-Net

## Abstract

**Background:**

To determine if a small number of annotated scans can enable clinically meaningful adaptation of a pre-trained convolutional neural network across different centers. Using non-contrast head CT brain extraction as the test case, we also sought to identify the factors contributing to performance drops when applying models to new sites.

**Materials and methods:**

Non-contrast head CT scans were used in this study, from two separate institutions (Center A: 595 scans; Center B: 486 scans). A public pre-trained U-Net model for brain tissue extraction was first evaluated on Center A dataset as a baseline. The model was then fine-tuned incrementally using stratified subsets of annotated scans (5, 15, 25, and 75). The pre-trained model was subsequently transferred to Center B dataset and adapted through the same incremental fine-tuning strategy. The primary metric of interest was the three-dimensional Dice similarity coefficient (DSC), with a pre-defined minimal important difference (MID) set at 0.01. To identify predictors of baseline DSC and fine-tuning gain driving cross-center performance degradation, variance inflation factor (VIF) screening and logit Gaussian linear models (GLMs) were applied, with SHapley Additive exPlanations (SHAP) analysis providing qualitative feature importance rankings.

**Results:**

At center A, the pre-trained model achieved a DSC of 0.9634 ± 0.0151. Fine-tuning with five annotated scans raised DSC to 0.9784 ± 0.0070 (*p* < 0.0001), exceeding the MID by 1.5-fold and reducing clinically unacceptable segmentations from 8.7 to 0.8%. A comparable gain was observed at Center B (*p* < 0.0001). Further increases to 15, 25, and 75 samples offered only marginal improvement below the MID threshold. Regression and SHAP analyses identified slice thickness as the dominant driver of both baseline performance degradation and fine-tuning improvement, accounting for 80–90% of predictive weight at both centers.

**Conclusion:**

For NCCT brain extraction assessed across two centers, fine-tuning a publicly pre-trained CNN using five local annotated scans can generate clinically valuable cross-center segmentation gains; slice thickness heterogeneity appears to be the dominant source of inter-center domain shift.

## 1 Introduction

Deep learning models for medical image segmentation have achieved expert-level performance in single-center evaluations (1). However, they often exhibit significant performance degradation when deployed to new clinical sites due to systematic differences in scanner hardware, acquisition protocols, and patient populations (1–3). This failure to generalize between centers is an ongoing obstacle to the extensive clinical use of automated segmentation methods, even for pre-trained models that are disseminated as open-source resources designed for wide deployment (4, 5). Many strategies have been proposed for domain adaptation (6), but the fundamental question of how few site-wise annotated samples are needed to achieve clinically meaningful adaptation and what specific dimensions of inter-center heterogeneity drive the domain gap has not been systematically addressed.

Non-contrast head CT (NCCT) brain extraction, often referred to as skull stripping, serves as an ideal task to study this issue. It is an essential preprocessing step in many quantitative analyses of neurological CT scans (7–9), such as brain volumetry, tissue segmentation, lesion quantification, and spatial normalization. This process is routinely performed at hospitals worldwide. The technical difficulty arises because the intensity values of brain parenchyma are very close to those of surrounding structures. This challenge is especially pronounced near the skull base, where thin bone lies adjacent to brain tissue and blood vessels (8, 10). Existing deep learning approaches have demonstrated strong single-center performance; for example, Akkus et al. (11) developed a deep learning-based CT brain extraction tool with publicly available pre-trained weights. Advanced architectures including nnU-Net, Swin UNETR, and TransUNet have further pushed segmentation boundaries. However, few studies systematically evaluate their cross-center adaptation capacity under limited local annotation resources (2, 12). As a result, the data efficiency of pre-trained medical segmentation models in heterogeneous multi-center settings remains poorly characterized.

Previous research in medical image segmentation indicates that obtaining satisfactory generalization often depends on training or adapting models using tens to hundreds of annotated samples (13), even when leveraging pre-trained models. For clinical centers, voxel-level segmentation of 3D CT or MRI volumes often demands considerable expert time for voxel-level 3D annotation, making routine deployment impractical (14, 15). Although several studies have examined how performance varies with sample size in organ segmentation (16–18), to the best of our knowledge, no study has systematically determined the minimum number of site-specific NCCT scans required for clinically meaningful brain extraction across centers or decomposed the mechanisms of cross-center performance degradation.

In this two-center retrospective study, we systematically varied the number of fine-tuning samples across NCCT scans from two hospitals, quantified the relative contribution of five heterogeneity dimensions to the domain gap through variance decomposition, and measured the sample efficiency gain conferred by pre-training versus training from scratch. We hypothesized that a well-pre-trained model can achieve operationally sufficient cross-center adaptation with far fewer samples than previously reported (≤15 scans), and that acquisition protocol parameters, rather than scanner brand or patient pathology, constitute the dominant source of cross-center performance variation. These findings may inform practical guidelines for deploying pre-trained segmentation models across heterogeneous clinical environments.

## 2 Materials and methods

### 2.1 Data acquisition

**Figure 1:**
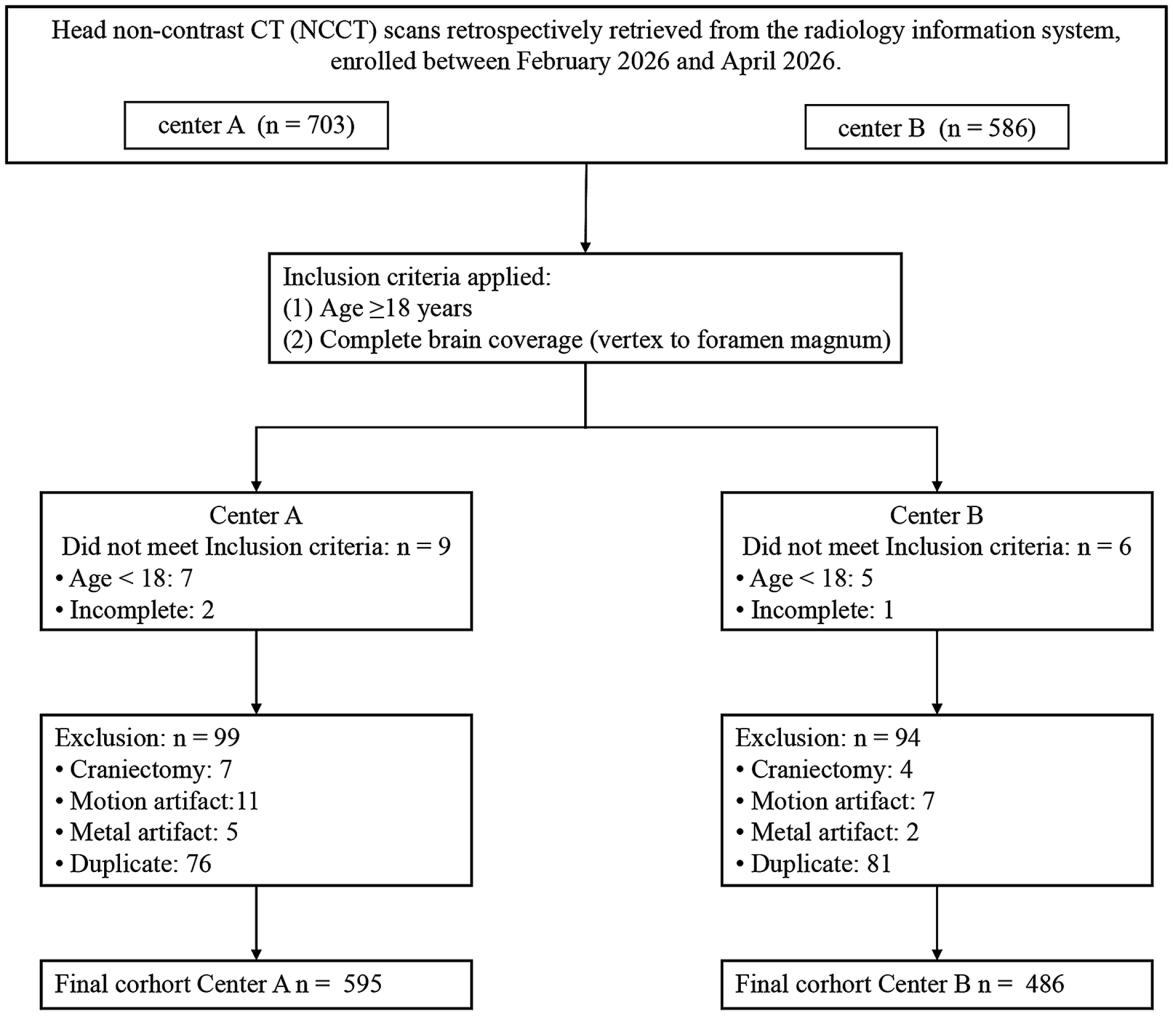
Flowchart of patient selection and scan inclusion. Overall, a total of 1,289 scans, including 703 from Center A and 586 from Center B, were screened between February 2026 and April 2026. After screening according to predefined inclusion and exclusion criteria, 595 eligible scans from Center A and 486 eligible scans from Center B were included in the final analysis.

This retrospective study was approved by the local institutional review board (NO.2024-KY-203 K) and complied with the Declaration of Helsinki. Written informed consent was waived, as only archived imaging data were retrospectively analyzed in this work. The data were collected from Liyang Hospital of Chinese Medicine and Shanghai Sixth People’s Hospital from February 2026 to April 2026 retrospectively. Inclusion criteria included: (1) patients aged 18 years or older who underwent head NCCT; (2) complete brain coverage from the vertex to the foramen magnum. Exclusion criteria were: (1) large cranial defects (e.g., post-decompressive craniectomy) precluding reliable delineation of brain boundaries; (2) severe motion artifacts; (3) metallic artifacts (e.g., aneurysm clips, deep brain stimulation electrodes) obscuring brain parenchyma; (4) duplicate scans from the same patient, only the initial examination was retained to maintain statistical independence. A summary of the inclusion and exclusion criteria is provided in Figure 1. Scanner protocols are detailed in Supplementary Table S1.

### 2.2 Ground-truth annotation

Ground-truth brain masks were manually delineated with ITK-SNAP (version 4.0.2) by a board-certified radiologist who has 8 years of neuroradiology experience. An attending neuroradiologist with 12 years’ experience then reviewed, refined, and confirmed all segmentations to ensure accuracy and consistency. These finalized masks were used as the reference standard for both model training and evaluation.

### 2.3 Pre-trained model selection

To simulate a clinically relevant scenario where a site uses a publicly available pre-trained model without retraining it locally, we selected the CT brain extraction model developed by Akkus et al. (11) as our baseline. This model was chosen because, to our knowledge, it is the only deep learning approach for NCCT brain extraction with both its architecture and pre-trained weights publicly accessible. Its training dataset consisted of stroke NCCT scans collected at Mayo Clinic, all acquired with 5 mm thick slices. This availability allows for reproducible evaluation without the variability introduced by re-implementation. The model’s U-Net architecture is also representative of the encoder–decoder frameworks commonly used in medical image segmentation, supporting the broader applicability of our findings.

The architecture follows the standard U-Net. All experiments reported here used the pre-trained weights published by Akkus et al. (11). The model and weights are available at https://github.com/aqqush/CT_BET.git.

### 2.4 Fine-tuning protocol and controls

#### 2.4.1 Data partitioning

Within each center, the dataset was randomly divided into a fixed training set comprising 75 scans, and a held-out test set (520 scans for Center A; 411 scans for Center B). The test sets were kept constant across all experiments to avoid bias in evaluation.

#### 2.4.2 Incremental fine-tuning

Two incremental fine-tuning pipelines were implemented: an independent initialization pipeline and the serial incremental pipeline, with the same stratified sampling strategy applied across all stages. The pre-trained baseline model (V1) was first evaluated on Center A data without any fine-tuning. For the serial pipeline, V2 to V5 were fine-tuned incrementally using stratified random samples of 5, 15, 25, and 75 scans from Center A, respectively. Each subsequent model was initialized from the best-performing weights of the previous fine-tuning stage. In the independent pipeline, V2 to V5 were independently fine-tuned from the original V1 weights using the same training subsets as in the serial pipeline, with each stage reinitialized from V1. For Center B, S1 represents the original pre-trained U-Net evaluated on the Center B dataset without local fine-tuning and serves as Center B′ s baseline. All S2 to S5 models from both pipelines were generated starting from raw pre-trained weights following the same sampling rules. Full-network fine-tuning was adopted. A fixed global random seed of 42 was applied uniformly for dataset splitting, model initialization.

#### 2.4.3 Sampling stability

To evaluate the effect of training sample selection, the 5-sample fine-tuning step was repeated using 10 distinct stratified random seeds at each center, ensuring non-overlapping samples.

#### 2.4.4 Pre-training dependence controls

Two control models were trained from scratch using random He-normal weight initialization. These models adopted the identical U-Net architecture and training protocol to quantify the contribution of pre-training to sample efficiency.

#### 2.4.5 Zero-annotation adaptation methods

Two adaptation approaches requiring no manual annotations from the target center were evaluated as comparators: test-time batch normalization adaptation and adversarial domain adaptation (AdvDA).

#### 2.4.6 Data augmentation and preprocessing controls

Training samples were augmented online with 0.8 probability by random rotation (±10°), scaling (±10%), translation (±5%), elastic deformation (*α* = 50, *σ* = 5), and Gaussian noise (σ = 0.01 × HU range). Spatial transforms were applied identically to images and labels, with nearest-neighbor interpolation for labels.

#### 2.4.7 Training configuration

All fine-tuning runs used the Adam optimizer with a learning rate of 1 × 10^−5^, selected via a fivefold cross-validation grid search. Learning-rate sensitivity was assessed across a one-order-of-magnitude range (5 × 10^−6^ to 5 × 10^−5^). Weighted categorical cross-entropy served as the loss function. Each training phase ran for 50 epochs (batch size = 8).

### 2.5 Evaluation metrics

The Dice Similarity Coefficient (DSC) was used to assess segmentation accuracy. Alongside statistical significance, we report absolute DSC differences (ΔDSC) to help interpret practical relevance. We used a threshold to distinguish mild suboptimal segmentation (DSC < 0.95) and severe unusable outputs (DSC < 0.90). Prior benchmarks have observed performance gaps of 0.01 to 0.03 between leading methods (19); accordingly, a threshold of ΔDSC ≥ 0.01 was pre-defined as the minimal important difference (MID) for pairwise comparisons.

### 2.6 Domain shift driver decomposition

To quantitatively identify imaging and clinical factors driving cross-center segmentation performance degradation, we adopted a two-stage analytical pipeline tailored to two core statistical challenges: DSC values represent proportional metrics bounded strictly between 0 and 1, and CT acquisition parameters may exhibit multicollinearity.

Variance Inflation Factor (VIF) screening was performed across all five candidate predictors (scanner manufacturer, mAs, slice thickness, field of view, and disease diagnosis). Predictors with VIF > 10 were defined as severely collinear and excluded from subsequent regression modeling to avoid biased estimation of factor effects. To accommodate the bounded distribution of DSC, we implemented logit transformation on raw DSC values prior to fitting Gaussian generalized linear models (GLMs). All input predictors were standardized ahead of model training to generate comparable standardized regression coefficients, which quantified the independent predictive weight of each imaging or clinical factor. Two separate regression models were constructed: one for baseline DSC (V1/S1) and another for fine-tuning segmentation gain.

As a model-agnostic supplementary analysis, SHapley Additive exPlanations (SHAP) were calculated from an XGBoost regression framework to provide qualitative rankings of feature importance.

### 2.7 Statistical analysis

Paired Wilcoxon signed-rank tests were conducted for all within-scan model comparisons, with Holm–Bonferroni correction applied to control for multiple comparisons at *α* = 0.05. Kruskal–Wallis tests and 95% confidence intervals of the coefficient of variation were used to quantify inter-seed variability across the ten-seed experiments. For stratified analyses, continuous acquisition parameters were dichotomized at clinically established thresholds (age: 60 years; slice thickness: 1 mm) or approximate sample medians (mAs: 250; FOV: 250). VIF multicollinearity screening was executed for all predictors. Beta regression was implemented to model bounded DSC outcomes, with standardized coefficients reported to compare factor contributions.

Mean absolute SHAP values derived from XGBoost served only for qualitative feature ranking; Spearman correlation was calculated to test whether the feature order from Beta regression aligned with SHAP-based ranking. All analyses were performed in Python 3.9.25.

## 3 Results

### 3.1 Patient characteristics

A total of 1,081 head NCCT scans were included in this study, comprising 595 scans from Center A and 486 scans from Center B. For each center, the dataset was split into a held-out test set (520 scans for Center A, 411 scans for Center B) and a 75-scan training set pool at each center for incremental fine-tuning. Substantial heterogeneity in scanning parameters was observed between the two centers, reflecting typical real-world clinical practice variations. Center A mainly used Philips scanners (79%) and Center B had a distribution of Siemens (36.5%), GE (31.9%), Philips (21.7%) and United Imaging Healthcare (9.5%). Differences in patient age, mAs, field of view, slice thickness, and diagnosis profiles were also observed. Each increment of training set data is randomly sampled from a fixed pool of 75 cases (Table 1).

**Table 1:** Detailed scanning parameters and clinical characteristics.

Variable	Center A					Center B				
	Test (*n* = 520)	Training (cumulative)				Test (*n* = 411)	Training (cumulative)			
		V2 (*n* = 5)	V3 (*n* = 15)	V4 (*n* = 25)	V5 (*n* = 75)		V2 (*n* = 5)	V3 (*n* = 15)	V4 (*n* = 25)	V5 (*n* = 75)
Age, years	58.2 ± 12.3	57.6 ± 13.1	58.5 ± 11.8	57.9 ± 12.5	58.1 ± 12.2	63.4 ± 13.0	62.8 ± 12.7	62.1 ± 13.2	60.5 ± 12.9	61.3 ± 13.1
Male, *n* (%)	286 (55.0)	3 (60.0)	5 (50.0)	7 (70.0)	27 (54.0)	222 (54.0)	3 (60.0)	4 (40.0)	5 (50.0)	26 (52.0)
Manufacturer										
GE	42	0	0	0	5	131	1	4	3	17
Philips	414	5	8	10	36	89	2	2	1	10
Siemens	36	0	2	0	6	150	2	3	6	19
UIH*	28	0	0	0	3	39	0	1	0	4
Age										
≤60, *n*	256	2	4	4	23	148	2	1	4	19
>60, *n*	264	3	6	6	27	263	3	6	6	31
mAs										
≤250, n	243	3	5	6	24	267	4	6	5	30
>250, *n*	277	2	5	4	26	144	1	4	5	20
FOV										
≤250, *n*	216	3	7	4	22	192	2	2	4	21
>250, *n*	304	2	3	6	28	219	3	8	6	29
Slice Thickness (mm)										
>1, *n*	285	3	7	6	29	209	3	7	5	26
≤1, *n*	235	2	3	4	21	202	2	3	5	24
Clinical diagnosis										
No intracranial abnormality	136	1	5	3	18	192	2	5	5	22
Neurological disease	384	4	5	6	32	219	3	5	5	28

Each included scan corresponded to a unique patient, as duplicate examinations from the same patient were excluded to maintain statistical independence. Training set values are cumulative totals at each fine-tuning stage. For example, the V3 model was fine-tuned using all samples from V2 plus the newly added batch, resulting in a cumulative total of 15 samples.

*UIH, United Imaging Healthcare.

### 3.2 Segmentation performance and sample-size saturation

Two fine-tuning pipelines, independent initialization and serial incremental fine-tuning were analyzed. The two pipelines achieved identical DSC values for the 5-scan group; minor performance gaps only appeared at sample sizes ≥ 15 due to cumulative weight inheritance bias in serial training.

**Figure 2:**
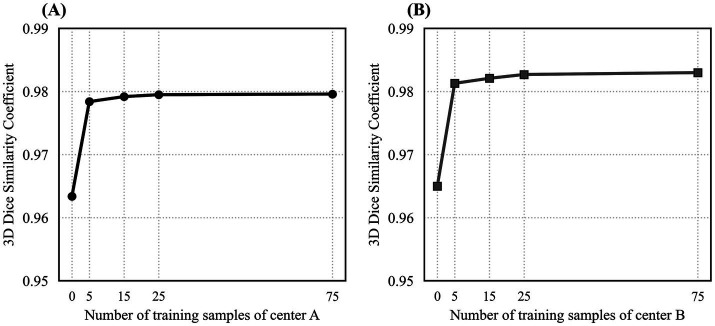
Segmentation performance curves of the independent initialization pipeline. **(A)** Center A; **(B)** Center B. The *x*-axis represents the quantity of annotated training scans, where 0 denotes the untuned pre-trained baseline V1/S1. The *y*-axis shows the mean 3D DSC values. Performance saturation is observed after fine-tuning with only 5 annotated scans.

The pre-trained U-Net (V1) achieved a mean 3D DSC of 0.9634 ± 0.0151 on the center A test set (*N* = 520). Despite the overall high accuracy, with 0.8% of scans showing severe segmentation failure (DSC < 0.90), 8.7% of scans still did not reach the clinically acceptable DSC threshold (≥ 0.95). Fine-tuning with 5 annotated samples raised performance to 0.9784 ± 0.0070, corresponding to an absolute gain of 0.0151 [95% CI: 0.0136, 0.0165] (corrected *p* < 0.0001). The improvement exceeds the predetermined MID by 1.5 times, and all cases with DSC below 0.90 have been eliminated. As the number of training samples increases, the performance improvement gradually weakens, and the increase does not reach the MID: the increase from 5 samples to 15 samples is 0.0015, and the increase from 25 to 75 samples is basically stagnant (Table 2 and Figure 2). Increasing sample size from 15 to 75 yielded only marginal improvements below the predetermined MID.

**Table 2:** Segmentation results of two fine-tuning pipelines.

Training phase	Center A – serial fine-tuning	Center A – independent initialization	Center B – serial fine-tuning	Center B – independent initialization
Baseline (V1/S1)	0.9634 ± 0.0151	0.9634 ± 0.0151	0.9650 ± 0.0132	0.9650 ± 0.0132
5 annotated scans	0.9784 ± 0.0070***	0.9784 ± 0.0070***	0.9813 ± 0.0078***	0.9813 ± 0.0078***
15 annotated scans	0.9799 ± 0.0068***	0.9792 ± 0.0069***	0.9831 ± 0.0065***	0.9821 ± 0.0067***
25 annotated scans	0.9801 ± 0.0051***	0.9795 ± 0.0062***	0.9846 ± 0.0055***	0.9827 ± 0.0059***
75 annotated scans	0.9801 ± 0.0070***	0.9797 ± 0.0068***	0.9851 ± 0.0052***	0.9830 ± 0.0054***

***Holm–Bonferroni corrected *p* ≤ 0.001 compared with the corresponding center baseline (V1 for Center A, S1 for Center B).

This saturation trend was consistent across Center B. Five-sample fine-tuning improved the cross-center baseline from 0.9650 ± 0.0132 to 0.9813 ± 0.0078 (ΔDSC = 0.0163, corrected *p* < 0.0001), exceeding the MID by a factor of 1.63, and further increases in sample size again showed diminishing returns below clinical relevance.

### 3.3 Validation and comparative experiments

Three control experiments were performed to test whether the 5-sample result was stable across training set compositions, dependent on pre-training, and superior to unsupervised alternatives.

A ten-seed sensitivity analysis using non-overlapping 5-sample training sets yielded a grand mean DSC of 0.9803 (95% CI: 0.9797, 0.9808) with a coefficient of variation of 1.79% (95% CI: 1.46, 2.13%). No significant variation was detected across seeds (Kruskal–Wallis *p* = 0.76), and the lowest-performing run (DSC = 0.9768) still exceeded the MID relative to baseline. Center B showed comparable stability (CV = 1.23%, Kruskal–Wallis *p* = 0.68).

To isolate the role of pre-training, we trained randomly initialized models under otherwise identical conditions. With 5 samples, the from-scratch model reached only 0.9098 ± 0.042 DSC, a deficit of 0.0703 compared with the fine-tuned model (corrected *p* < 0.0001). When given the full 75-sample training set, the from-scratch model improved to 0.9732 ± 0.015 but still fell short of the 5-sample fine-tuned result (difference of 0.0052, corrected *p* < 0.0001). Put differently, the pre-trained initialization compensated for more than 70 annotated samples, and the observed sample efficiency cannot be achieved without it. Results at Center B were consistent (both corrected *p* < 0.0001).

Two unsupervised domain adaptation methods requiring no local annotations were further compared. Test-time batch normalization adaptation recovered 18% of the cross-center performance gap (DSC = 0.9682 ± 0.0105), while adversarial domain adaptation recovered 34% (DSC = 0.9706 ± 0.0098). The better-performing adversarial domain adaptation still yielded a DSC score 0.0107 lower than the 5-sample fine-tuned model (corrected *p* < 0.0001).

### 3.4 Domain shift driver decomposition

Prior to regression modeling, VIF multicollinearity screening was performed for both centers, yielding maximum VIF values of 4.51 (Center A) and 5.61 (Center B). All values were well below the threshold of 10, indicating no severe collinearity; thus, all five imaging and clinical predictors were retained for subsequent logit-transformed Gaussian GLM analysis.

Standardized GLM coefficients identified slice thickness as the dominant predictor of baseline segmentation performance across both centers. Thin-slice scans exhibited significantly lower baseline DSC than thick-slice scans at Center A (*β* = −0.181, *p* < 0.001) and Center B (*β* = −0.198, p < 0.001) (Table 3). Clinical diagnosis served as the second significant predictor (Center A: *β* = −0.053, p < 0.001; Center B: *β* = −0.030, *p* = 0.0065), while scanner manufacturer, radiation dose, and FOV showed negligible, non-significant effects at both sites (all |*β*| < 0.02, *p* > 0.05). Subgroup analyses confirmed this domain-shift pattern: thin-slice scans presented consistently poorer baseline accuracy (Center A: 0.9553 ± 0.0189; Center B: 0.9546 ± 0.0175) relative to thick-slice protocols (Center A: 0.9700 ± 0.0052; Center B: 0.9703 ± 0.0048, all *p* < 0.001), consistent with the thick-slice-dominated source dataset of the pre-trained model.

**Table 3:** Cross-center decomposition of baseline DSC and fine-tuning gain using logit GLM and SHAP analysis.

Factor	Center A				Center B			
	Baseline Std *β*	ΔDSC Std *β*	SHAP V1 (%)	SHAP ΔDSC (%)	Baseline Std *β*	ΔDSC Std *β*	SHAP V1 (%)	SHAP ΔDSC (%)
Slice thickness	−0.181***	0.011***	79.6	85.9	−0.198***	0.012***	83.6	90.0
Clinical diagnosis	−0.053***	0.001**	7.9	4.6	−0.030**	0.001	5.1	1.6
Manufacturer	0.001	0.002***	3.4	4.4	0.016	−0.001	3.6	2.8
Radiation mAs dose	0.005	0.001	5.3	3.1	0.009	0.001	4.9	3.6
FOV	0.013	0.001	3.8	2.0	0.010	0.000	2.8	1.9

Std β, standardized logit GLM coefficient; ΔDSC = V2/S2 − V1/S1 fine-tuning improvement. SHAP values represent normalized relative feature importance. Slice thickness: thin ≤1 mm, thick >1 mm; radiation dose: low ≤ 250 mAs, high ≥ 250 mAs; FOV: small ≤ 250 mm, large ≥ 250 mm; Manufacturer: four levels (Philips, Siemens, GE, UIH); Clinical diagnosis: healthy, pathology. Significance: *p < 0.05, **p < 0.01, ***p < 0.001; unmarked terms non-significant. All predictors exhibited VIF < 10 with no severe multicollinearity.

When modeling fine-tuning improvement, slice thickness remained the primary driver of segmentation gain at both centers (Center A: *β* = 0.011, *p* < 0.001; Center B: *β* = 0.012, *p* < 0.001), indicating prominent accuracy recovery for thin-slice scans after few-shot fine-tuning. Stratified results revealed minimal gains in thick-slice cases (Center A: 0.0055; Center B: 0.0059) versus substantial improvements in thin-slice cases (Center A: 0.0267; Center B: 0.0283). Manufacturer significantly predicted fine-tuning benefit at Center A (*β* = 0.002, *p* < 0.001) but showed no significant effect at Center B (*β* = −0.001, *p* = 0.0737). Diagnosis exerted a minor positive effect at Center A (*β* = 0.001, *p* = 0.0099) and no effect at Center B, while dose and FOV contributed negligibly at both centers.

SHAP analysis consistently prioritized slice thickness as the most influential feature, accounting for 79.6% (Center A) and 83.6% (Center B) of baseline predictive importance, as well as 85.9 and 90.0% of ΔDSC predictive importance, respectively. Perfect rank concordance existed between GLM coefficients and SHAP feature importance (*p* < 0.001) at both centers for baseline and improvement models. A strong negative correlation between baseline DSC and fine-tuning gain was observed in both cohorts (Center A: *r* = −0.909; Center B: *r* = −0.924; all *p* < 0.001), demonstrating that domain-shift-impaired cases achieved the largest accuracy restoration via few-shot adaptation.

### 3.5 Control experiments and robustness

Data augmentation yielded modest yet consistent DSC improvements (mean gain 0.0010 ± 0.0003) across all training set sizes ranging from 15 to 75 samples. No significant interaction was found between augmentation and sample size (two-way aligned rank transform ANOVA, corrected *p* = 0.763), suggesting these effects are additive rather than overlapping.

Learning rate sensitivity analysis demonstrated stable fine-tuning across a one order of magnitude range from 5 × 10^−6^ to 5 × 10^−5^, with DSC variation under 0.003 using 5 samples, and all tested rates achieving improvements beyond the MID. Instability only arose at a much higher learning rate of 1 × 10^−4^.

Finally, pairwise comparisons between models highlighted a saturation point in performance. While 5-sample fine-tuning significantly outperformed baseline and 15-sample fine-tuning was better than 5-sample, no significant difference was observed between the 15-sample and 75-sample models (corrected *p* = 0.3764). These data demonstrate performance plateaus once more than 15 annotated scans are used for fine-tuning.

### 3.6 Segmentation error visualization

**Figure 3:**
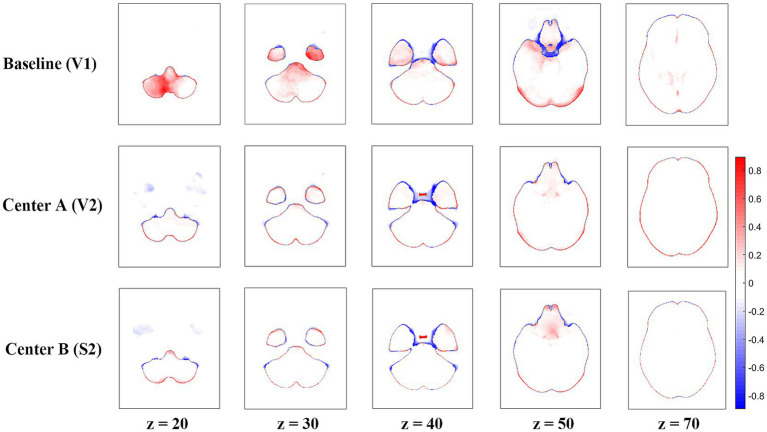
Spatial segmentation error heatmaps in MNI152 space. Representative axial slices at *Z* = 20, 30, 40, 50, and 70. Row 1: Baseline model (V1), Center A; Row 2: 5-sample fine-tuned model (V2), Center A; Row 3: 5-sample fine-tuned model (S2), Center B. Red indicates under-segmentation; Blue indicates over-segmentation; Color intensity reflects error magnitude.

To identify systematic segmentation errors, voxel-wise error maps from Center A and Center B were registered to the MNI152 standard space and averaged to create spatial heatmaps (Figure 3). Each heatmap encodes two dimensions of segmentation error via color: red regions mark under-segmentation, blue regions mark over-segmentation, and color saturation scales with the magnitude of voxel-wise error.

Errors concentrated at the brain periphery and skull base, while central regions and slices superior to the third ventricle showed minimal deviation. This spatial pattern was consistent across both centers. Five-sample fine-tuning reduced peripheral under-segmentation relative to the untuned baseline (V1 vs. V2, Figure 3, rows 1–2), with the most pronounced improvement observed at the skull base margins. S2 had a similar error distribution to V2, and no new systematic error regions were added by cross-center transfer (Figure 3, row 3).

After fine-tuning, V2 achieved a mean error of 3.2 ± 1.5 mm with a maximum error of 8.7 mm and 89.6% of voxels within 5 mm. S2 showed similar performance, with a mean error of 3.3 ± 1.6 mm, a maximum error of 8.9 mm, and 88.9% of voxels within 5 mm. No voxel exceeded a 9 mm deviation at either center.

## 4 Discussion

In this two-center retrospective study, we demonstrated that a well-pre-trained CT brain extraction model achieves effective cross-center adaptation with as few as five site-specific annotated scans—far below the on-the-order-of tens to scores of annotated cases commonly assumed in prior cross-center or cross-modality segmentation studies (17, 18, 20).

One contributing factor to this sample efficiency is the quality of pre-training: a randomly initialized model trained on 75 samples still underperformed the 5-sample fine-tuned pre-trained model. This finding is in line with theoretical and empirical studies showing that well-pre-trained models can achieve a given target performance with substantially fewer target-domain examples than training from scratch by reusing and lightly recalibrating existing feature representations rather than learning them *de novo* (18).

Our variance decomposition analysis provides, to our knowledge, the first quantitative attribution of cross-center performance degradation in CT brain extraction to specific imaging protocol dimensions. Logit-transformed Gaussian GLM and complementary SHAP analyses consistently identified slice thickness as the overwhelmingly dominant predictor of both baseline segmentation failure and few-shot fine-tuning gains across two independent patient cohorts. This finding is consistent with prior work (21, 22) showing that slice thickness and associated longitudinal partial volume effects substantially alter the geometric fidelity and contrast of thin anatomical structures in CT, particularly at bone–soft tissue interfaces. Similarly, Chen et al. (3) observed that prostate auto-segmentation models trained predominantly on 3 mm CT slices degraded when deployed on 2 mm acquisitions, attributing this not to image quality but to distribution mismatch in slice-thickness-specific learned features. Our findings extend this mechanism to brain CT: partial volume averaging at bone–brain interfaces varies systematically with slice thickness, producing appearance shifts beyond the learned feature space of models trained on narrower protocol distributions. The observation that thin-slice scans exhibited both the lowest baseline performance and the greatest fine-tuning benefit (Center A: ΔDSC = 0.0267 thin-slice vs. 0.0055 thick-slice; Center B: ΔDSC = 0.0283 thin-slice vs. 0.0059 for thick-slice) implies that the original training dataset of Akkus et al. (11) was predominantly composed of thick-slice acquisitions, creating a systematic domain gap for thin-slice protocols. Radiation dose and reconstruction FOV did not significantly affect performance, indicating that these routine protocol variations are already well-represented in the pre-training distribution.

Annotation-free domain adaptation methods, including test-time batch normalization adaptation and adversarial domain adaptation, recovered only 18 and 34% of the cross-center performance gap, respectively—both significantly inferior to 5-sample supervised fine-tuning. This result contrasts with reports in MRI segmentation, where unsupervised or source-free domain adaptation and intensity standardization have achieved substantial performance gains across sites and modalities (23). The limited effectiveness of unsupervised methods in our setting partly reflects the geometric nature of the CT domain gap, where differences in slice thickness change effective spatial resolution and boundary sharpness in 3D in ways that are not addressed by intensity normalization alone (18, 23, 24).

These results have direct implications for the clinical deployment of automated CT brain extraction tools. First, our results are consistent with prior interactive and few-shot segmentation frameworks, confirming that a handful of annotated images can yield substantial performance gains when combined with strong pre-training. Second, our variance decomposition results indicate that data curation strategies for pre-training should emphasize slice thickness diversity rather than scanner or manufacturer diversity—an insight that may inform future training dataset design. Finally, the complete eradication of severe segmentation failures (DSC < 0.90) after 5-sample fine-tuning solves a major safety concern of unsupervised clinical deployment where catastrophic errors, however infrequent, can erode clinician trust in automated tools.

This study has several limitations. First, only two centers were included in this retrospective analysis. Although the consistent results from both hospitals support partial generalizability, additional multi-center cohorts covering more scanner vendors, patient demographics and geographic backgrounds are required to further verify our findings, which we plan to explore in follow-up research. Second, our analysis was limited to a single architecture (U-Net) with one set of publicly available pre-trained weights; the optimal minimal fine-tuning sample size may vary for transformer-based segmentation models, 3D volumetric networks or alternative pre-training paradigms. Third, individual-level anatomical factors and unmeasured confounders contribute to segmentation variability; future work incorporating patient-level anatomical metrics (e.g., brain atrophy grade, ethnicity) may improve explanatory power.

In conclusion, five annotated site-specific scans are sufficient for effective cross-center adaptation of a pre-trained CT brain extraction model, with slice thickness heterogeneity identified as the primary driver of the domain gap. By addressing multi-site generalization challenges with minimal annotation burden, this strategy enables scalable deployment of automated brain extraction across diverse clinical neuroradiology centers.

## Data Availability

The raw data supporting the conclusions of this article will be made available by the authors, without undue reservation.
