# Supplementary material for: Data-centric strategies enable cross-center generalization of convolutional neural networks for CT brain extraction
Source: Front Neurol. 2026 Aug 3;17:1895626. doi: 10.3389/fneur.2026.1895626 (PMC13477930; doi:10.3389/fneur.2026.1895626)
Supplement: Supplementary file 1 [file Table_1.DOCX]

Supplementary Material

**Table S1. Imaging Protocol and Parameters in Different Manufacturers for multi-modal CT Images**

| **Centers** | **CT equipment** | **NCCT** |
| --- | --- | --- |
| Center A | UIH  uCT 960PLUS | Tube voltage: 120 kV; tube current: 450–500 mA; reconstruction thickness: 1.0 mm; 5 mm |
| Center A | SIEMENS  SOMATOM Definition Flash | Tube voltage: 120 kV; tube current: 160–250 mA; reconstruction thickness: 1.0 mm; 1.5mm; 5 mm |
| Center A | Philips Brilliance iCT 256 | Tube voltage: 120 kV; tube current: 250–300 mA; reconstruction thickness: 4–5 mm; 1mm |
| Center A | GE Revolution | Tube voltage: 120 kV; tube current: 270–350 mA; reconstruction thickness: 1.0 mm; 5 mm |
| Center B | SIEMENS  SOMATOM Definition Flash | Tube voltage: 120 kV; tube current: 160–250 mA; reconstruction thickness: 5.0 mm; 1mm |
| Center B | Philips Brilliance iCT 256 | Tube voltage: 120 kV; tube current: 250–300 mA; reconstruction thickness: 4–5 mm; 1mm |
| Center B | GE Revolution | Tube voltage: 120 kV; tube current: 270–350 mA; reconstruction thickness: 1.0 mm; 5 mm |
| Center B | UIH  uCT 780 | Tube voltage: 120 kV; tube current: 450–500 mA; reconstruction thickness: 0.625-1.0 mm; 5 mm |

**Table S2. Stratified performance by key parameters**

|  | V1 | V2 | p-value | S1 | S2 | p-value |
| --- | --- | --- | --- | --- | --- | --- |
| ****Manufacturer**** |  |  |  |  |  |  |
| Philips | 0.9641 | 0.9790 | <0.001 | 0.9690 | 0.9828 | <0.001 |
| Siemens | 0.9542 | 0.9790 | <0.001 | 0.9586 | 0.9832 | <0.001 |
| GE | 0.9626 | 0.9725 | 0.02 | 0.9661 | 0.9763 | <0.001 |
| UIH | 0.9648 | 0.9781 | 0.03 | 0.9695 | 0.9820 | 0.01 |
| **Age (years)** |  |  |  |  |  |  |
| ≤60 | 0.9628 | 0.9744 | <0.001 | 0.9671 | 0.9787 | <0.001 |
| ＞60 | 0.9644 | 0.9737 | <0.001 | 0.9693 | 0.9771 | <0.001 |
| **mAs** |  |  |  |  |  |  |
| ≤250 | 0.9663 | 0.9776 | <0.001 | 0.9710 | 0.9815 | <0.001 |
| ＞250 | 0.9617 | 0.9789 | <0.001 | 0.9654 | 0.9823 | <0.001 |
| **Slice Thickness** |  |  |  |  |  |  |
| >1mm | 0.9700 | 0.9820 | <0.001 | 0.9746 | 0.9820 | <0.001 |
| ≤1mm | 0.9553 | 0.9755 | <0.001 | 0.9546 | 0.9703 | <0.001 |
| **FOV** |  |  |  |  |  |  |
| ≤250 | 0.9646 | 0.9785 | <0.001 | 0.9698 | 0.9819 | <0.001 |
| ＞250 | 0.9625 | 0.9784 | <0.001 | 0.9662 | 0.9811 | <0.001 |
| **Clinical diagnosis** |  |  |  |  |  |  |
| No intracranial abnormality | 0.9678 | 0.9791 | <0.001 | 0.9726 | 0.9835 | <0.001 |
| Neurological disease | 0.9618 | 0.9782 | <0.001 | 0.9657 | 0.9826 | <0.001 |
